# Supplementary material for: Against the Odds: Hybrid Zones between Mangrove Killifish Species with Different Mating Systems
Source: Genes (Basel). 2021 Sep 24;12(10):1486. doi: 10.3390/genes12101486 (PMC8535463; doi:10.3390/genes12101486)
Supplement: Supplementary file 1 [file genes-12-01486-s001.zip › genes-1363723 supplymentary/supplementary.pdf]

Supporting information for

Against the odds: recent hybrid zones between mangrove killifish species with different mating systems

Waldir M. Berbel-Filho[1,2]*, Andrey Tatarenkov[3], George Pacheco[4], Helder M. V. Espírito-Santo[5], Mateus G. Lira[6], Carlos Garcia de Leaniz[2], John C. Avise[3], Sergio M. Q. Lima[6], Carlos M. Rodríguez-López[7], and Sofia Consuegra[2]*

1 Department of Biology, University of Oklahoma, Norman, OK, USA. (present address)

2 Department of Biosciences, College of Science, Swansea University, Swansea, UK.

3 Department of Ecology and Evolutionary Biology, University of California, Irvine, USA.

4 Section for Evolutionary Genomics, The GLOBE Institute, Faculty of Health and Medical Sciences, University of Copenhagen, 1353 Copenhagen, Denmark.

5 Núcleo de Ecologia Aquática e Pesca da Amazônia, Universidade Federal do Pará, Belém, Brazil.

6 Laboratório de Ictiologia Sistemática e Evolutiva, Departamento de Botânica e Zoologia, Universidade Federal do Rio Grande, Natal, Brazil.

7 Environmental Epigenetics and Genetics Group, Department of Horticulture, College of Agriculture, Food and Environment, University of Kentucky, Lexington, KY, USA.

*Corresponding authors: waldirmbf@gmail.com; s.consuegra@swansea.ac.uk

Library preparation

200ng of genomic DNA were digested using a *Eco*RI (cutsite: GAATTC) and *Hpa*II (cutsite: CCGG). Digested DNA was ligated to individually barcoded adapters with a *Hpa*II cut site overhang and a common *Eco*RI Y adapter. Ligation products were individually cleaned to remove excess of adapters using Agencourt AMPure XP purification system (#A63880, Beckman Coulter, Brea, CA, USA) at a v/v ratio of 0.85 following the manufacturer's instructions. A single library was produced by pooling 20ng of DNA from each sample and amplified in eight separate PCR reactions (25 µL each), containing 10 µL of library DNA, 5 µL of Q5 high fidelity buffer, 0.25 µL of Q5 high fidelity polymerase, and 1 µL of each Forward and Reverse common primer at 10 µM, 0.5 µL of 10 µM dNTP and 7.25 µL of pure sterile water. PCR products were pooled after amplification and size-selected (range 200 – 350 bp) using Agencourt AMPure XP magnetic beads. The size-selected library was sequenced in an Illumina NextSeq500 sequencer platform (Cardiff University Genomics Research Hub) to obtain 125bp paired-end reads.

Data processing

Paired-end reads were processed using a combination of packages in the Linux bash shell environment. First, we used GBSX v 1.3 [1] to demultiplex the paired-end reads allowing for one mismatch in the barcodes (-mb 1), no mismatch in the enzyme cut-site (-me 0), and ensuring that no common sequencing adapter was removed (-ca false). We then filtered (-qtrim r; -minlength 25) and merged the reads by individual using BBmap tools [2] mapped to *Kryptolebias marmoratus* reference genome (assembly size = 680.3Mb; number of scaffolds = 3,073; N50 = 2,229,659; GC content = 37.76%; Rhee, et al. 2017) using Bowtie 2 v. 2.2.3 with default parameters [3] and generated filtered and indexed individual BAM files with SAMtools v. 1.9 [4].

To call genotypes, we used ANGSD v 0.9.2.9 [5] with the following parameters: minimum mapping quality (-minMapQ 30), minimum base quality (-minQ 20), only 5% of missing data to consider a site or SNP -minInd 95%), maximum of 500 reads per position per individual (-setMaxDepth 500X per individual), minimum genotype posterior probability (-postCutoff 0.95). Single and double-tons (SNPs that only appeared in one or two individuals) were removed based on minimum minor allele frequencies (-MinMaf). Anomalous reads were filtered according to the flags (-remove_bads 1; SAM flag above 255). Adjusted mapping quality for excessive mismatches was done using (-C 50). BAQ computation was performed (-baq 1), and the minimum coverage for genotype calling was 3 (-geno_minDepth 3). We used SAMtools genotype likelihood model (-GL 1) and estimated posterior genotype probabilities assuming a uniform prior (-doPost 2). In addition, we used the ANGSD SNP calling method (-SNP_pval 1e-6), where a Likelihood Ratio Test is used to compare between the null (maf = 0) and alternative (estimated maf) hypotheses by using a X^2 distribution with one degree of freedom.

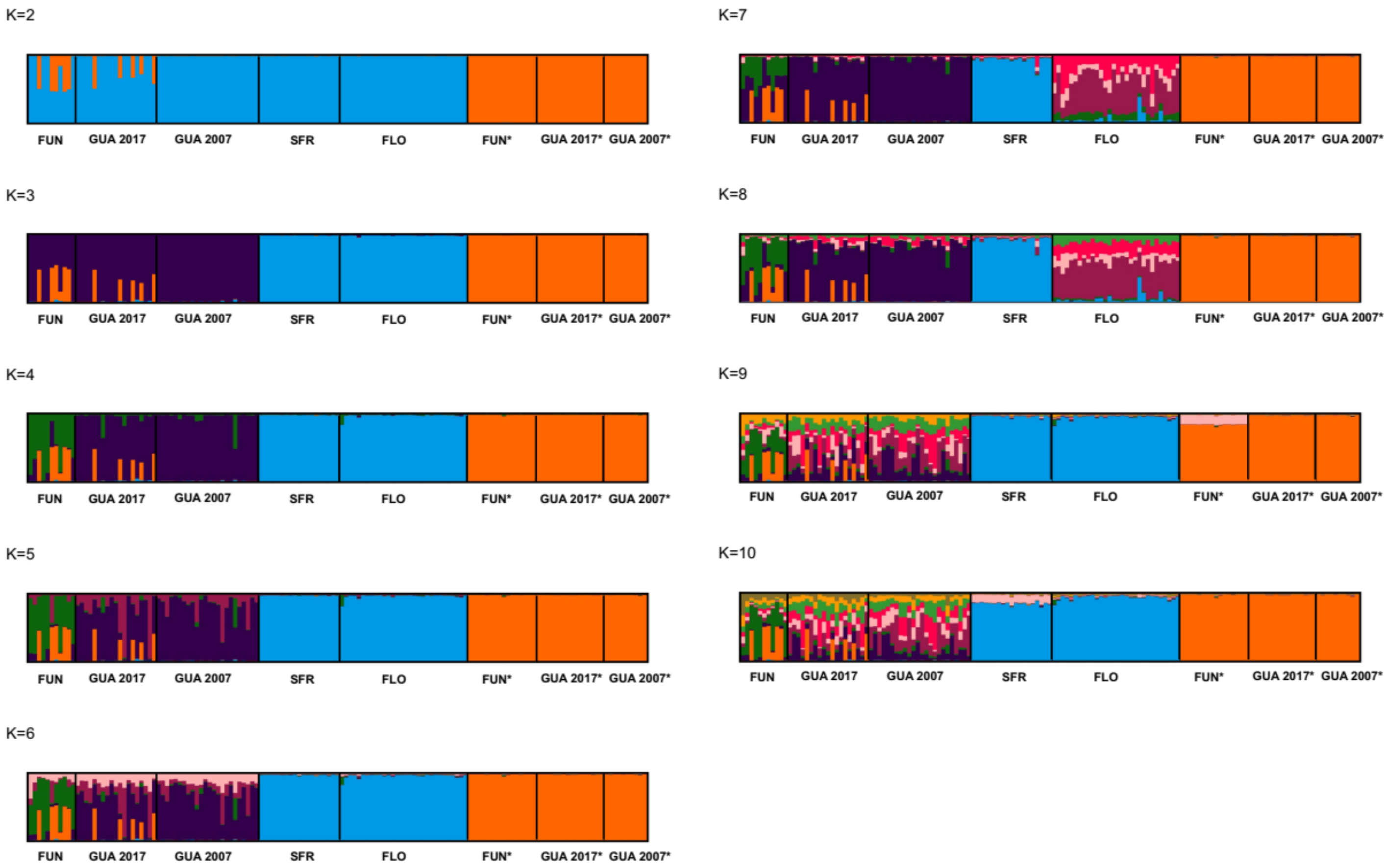

Figure S1. Individual ancestry plots with the highest likelihood runs for each K value ran in STRUCTURE with genotypes for 16 microsatellites for 103 *K. ocellatus* and 42 *K. hermaphroditus* individuals. Asterisk denotes locations for individuals with *K. hermaphroditus* mtDNA.

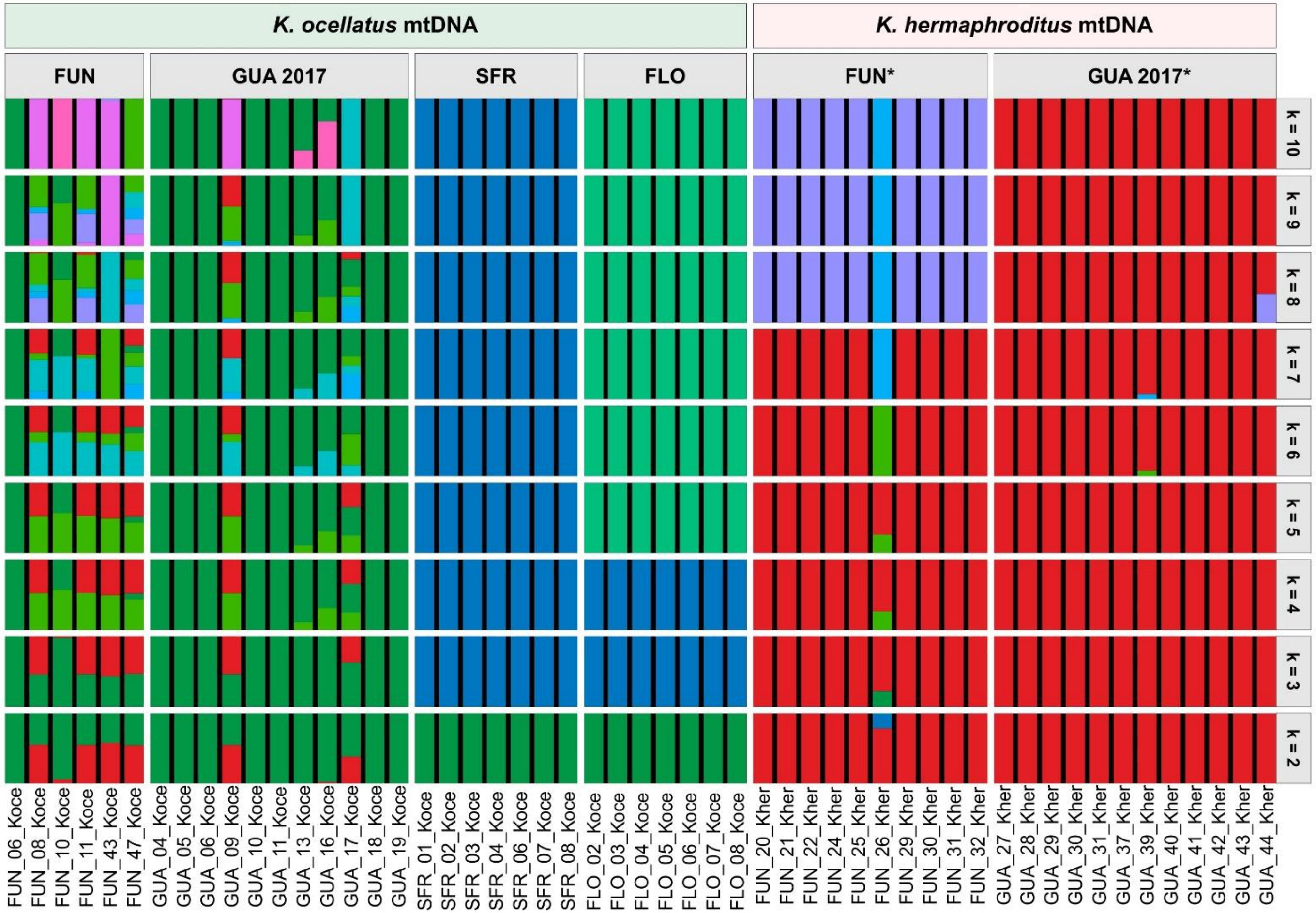

Figure S2. Individual ancestry plots with each K value ran in nsgAdmix with 5,477 SNPs from 31 *K. ocellatus* individuals and 22 *K. hermaphroditus* individuals. Asterisk denotes locations for individuals with *K. hermaphroditus* mtDNA.

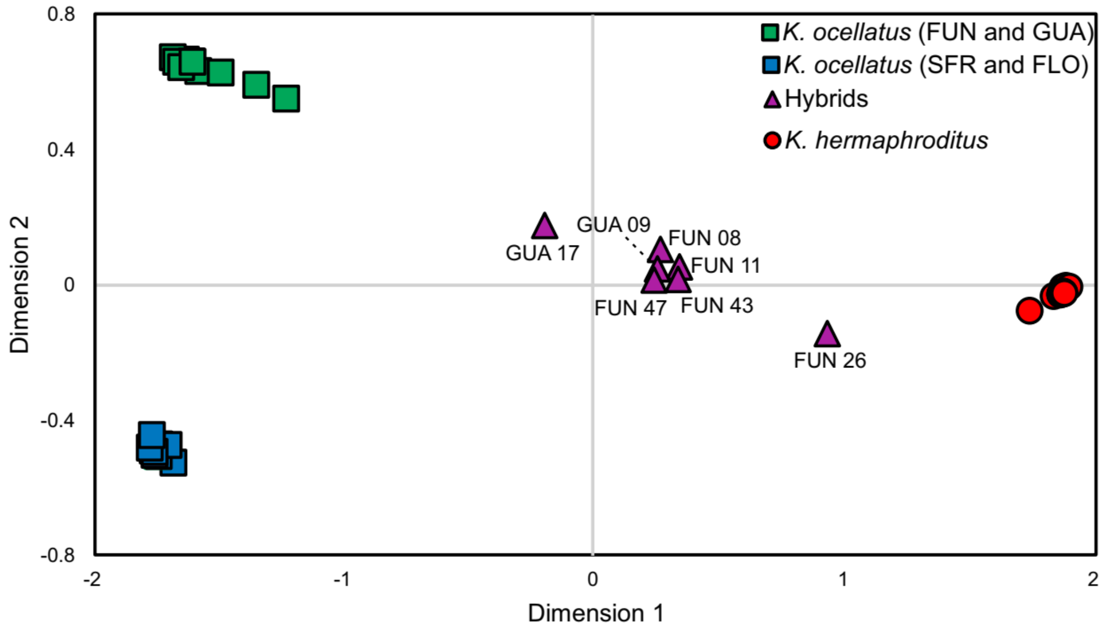

Figure S3. Multidimensional Scaling (MDS) based on the genetic distances from 5,477 SNPs. Hybrid individuals (see results) are highlighted with their respective labels.

Supplementary Table S1. Summary of 58 samples sequenced in the genotype-by-sequencing (msGBS) library. Individuals in red failed to pass the filtering cutoff (≥500kb reads). Superscript letters on ID denotes F1 and backcrosses.Parameters 'proportion of heterozygous sites' (for Dataset I), 'coverage and missing data' (for Dataset II) are described in Material and Methods.

ID	Species (identified using *cox1*)	Sampling location	Number of reads	Uniquely mapped (%)	Coverage (X)	Missing data (%)	Proportion of Heterozygous sites
GUA_27	*K. hermaphroditus*	GUA	8,962,202	90.30	250.4	0.00	0.03
GUA_28	*K. hermaphroditus*	GUA	4,161,657	90.87	114.9	0.02	0.03
GUA_29	*K. hermaphroditus*	GUA	13,831,746	90.29	380.1	0.00	0.02
GUA_30	*K. hermaphroditus*	GUA	7,000,238	90.05	190.2	0.00	0.02
GUA_31	*K. hermaphroditus*	GUA	6,646,432	89.95	185.2	0.02	0.03
GUA_37	*K. hermaphroditus*	GUA	3,013,973	90.06	82.2	0.18	0.03
GUA_39	*K. hermaphroditus*	GUA	3,472,656	89.73	93.5	0.02	0.04
GUA_40	*K. hermaphroditus*	GUA	13,651,662	90.50	382.6	0.00	0.03
GUA_41	*K. hermaphroditus*	GUA	5,348,736	90.08	147.3	0.04	0.03
GUA_42	*K. hermaphroditus*	GUA	1,786,528	91.00	48.8	0.20	0.02
GUA_43	*K. hermaphroditus*	GUA	7,269,128	89.39	197.0	0.00	0.03
GUA_44	*K. hermaphroditus*	GUA	7,937,202	89.92	216.6	0.00	0.02

FUN_20	*K. hermaphroditus*	FUN	8,493,250	89.83	229.9	0.00	0.02
FUN_21	*K. hermaphroditus*	FUN	4,140,936	90.70	113.3	0.00	0.03
FUN_22	*K. hermaphroditus*	FUN	4,990,777	90.96	138.7	0.00	0.03
FUN_24	*K. hermaphroditus*	FUN	3,520,363	89.86	98.6	0.02	0.03
FUN_25	*K. hermaphroditus*	FUN	4,421,739	90.34	120.7	0.04	0.03
FUN_26[Ba]	*K. hermaphroditus*	FUN	1,361,947	69.56	24.9	0.84	0.32
FUN_29	*K. hermaphroditus*	FUN	7,611,704	89.30	208.2	0.00	0.02
FUN_30	*K. hermaphroditus*	FUN	2,892,977	88.63	77.2	0.00	0.03
FUN_31	*K. hermaphroditus*	FUN	9,160,363	89.53	251.5	0.00	0.03
FUN_32	*K. hermaphroditus*	FUN	2,322,888	88.84	62.4	0.02	0.02
GUA_04	*K. ocellatus*	GUA	5,033,372	77.40	102.4	0.15	0.08
GUA_05	*K. ocellatus*	GUA	11,979935	75.68	235.1	0.00	0.08
GUA_06	*K. ocellatus*	GUA	6,418,126	81.00	143.5	0.04	0.08
GUA_09[F1]	*K. ocellatus*	GUA	5,784,849	84.54	138.1	0.05	0.61
GUA_10	*K. ocellatus*	GUA	11,097,202	81.18	243.2	0.04	0.08
GUA_11	*K. ocellatus*	GUA	2,208,760	75.05	44.5	0.46	0.06
GUA_13	*K. ocellatus*	GUA	11,446,137	77.19	230.1	0.00	0.09
GUA_16	*K. ocellatus*	GUA	14,282,577	80.18	313.2	0.00	0.11
GUA_17[Ba]	*K. ocellatus*	GUA	4,522,779	72.17	91.6	0.00	0.50

GUA_18	*K. ocellatus*	GUA	7,266,866	79.34	149.5	0.05	0.08
GUA_19	*K. ocellatus*	GUA	8,150,733	81.55	177.2	0.09	0.07
GUA_20[Ba]	*K. ocellatus*	GUA	20,647	NA	NA	NA	NA
GUA_24[Ba]	*K. ocellatus*	GUA	4,778	NA	NA	NA	NA
GUA_62[F1]	*K. ocellatus*	GUA	300,213	NA	NA	NA	NA
FUN_06	*K. ocellatus*	FUN	5,098,517	81.78	111.7	0.26	0.06
FUN_08[F1]	*K. ocellatus*	FUN	9,433,950	86.75	234.2	0.00	0.60
FUN_10	*K. ocellatus*	FUN	6,856,002	81.10	145.5	0.02	0.14
FUN_11[F1]	*K. ocellatus*	FUN	7,773,860	86.68	199.0	0.00	0.62
FUN_13[F1]	*K. ocellatus*	FUN	2,645	NA	NA	NA	NA
FUN_41[Ba]	*K. ocellatus*	FUN	7,793	NA	NA	NA	NA
FUN_43[F1]	*K. ocellatus*	FUN	578,575	83.31	13.8	4.14	0.48
FUN_47[F1]	*K. ocellatus*	FUN	564,546	78.82	12.4	4.16	0.50
FLO_02	*K. ocellatus*	FLO	13,605,858	81.73	301.5	0.04	0.05
FLO_03	*K. ocellatus*	FLO	2,439,264	70.34	48.0	0.38	0.06
FLO_04	*K. ocellatus*	FLO	2,167,505	79.99	46.8	0.40	0.06
FLO_05	*K. ocellatus*	FLO	2,517,261	81.12	53.6	0.66	0.06
FLO_06	*K. ocellatus*	FLO	4,609,454	78.83	96.2	0.11	0.06
FLO_07	*K. ocellatus*	FLO	4,620,891	81.46	96.3	0.02	0.05

FLO_08	*K. ocellatus*	FLO	6,823,947	79.43	149.9	0.00	0.06
SFR_01	*K. ocellatus*	SFR	9,375,699	81.74	209.4	0.05	0.06
SFR_02	*K. ocellatus*	SFR	2,671,245	82.54	60.3	0.37	0.06
SFR_03	*K. ocellatus*	SFR	8,014,672	81.64	177.9	0.11	0.06
SFR_04	*K. ocellatus*	SFR	8,136,965	83.09	177.8	0.00	0.07
SFR_06	*K. ocellatus*	SFR	10,875,988	82.37	240.8	0.15	0.05
SFR_07	*K. ocellatus*	SFR	10,572775	82.07	235.7	0.05	0.06
SFR_08	*K. ocellatus*	SFR	684,671	77.07	14.5	4.93	0.05
**Average**			**6,370,039**	**83.90**	**152.98**	**0.34**	**0.11**

Supplementary Table S2. Descriptive statistics of genetic variation at microsatellite loci in (*a*) 103 *Kryptolebias ocellatus* and (*b*) 42 *K. hermaphroditus* individuals. N = sample size; P_{99} = proportion of polymorphic loci (99% criterion); A = average number of alleles; H_E = expected heterozygosity; H_O = observed heterozygosity. Sampling locations are described in Table 1.

**Sampling location**	***N***	***P*₉₉**	***A***	***H*E**	***H*O**
(a) Individuals with *K. ocellatus* mtDNA					
GUA 2007	24	0.93	8.19	0.60	0.53
GUA 2017	19	1.00	9.31	0.65	0.64
FUN 2017	11	0.93	6.56	0.66	0.70
SFR	19	0.87	7.25	0.57	0.63
FLO	30	0.81	9.88	0.54	0.52
**Mean**	**20.6**	**0.90**	**3.83**	**0.60**	**0.60**
(b) Individuals with *K. hermaphroditus* mtDNA					
GUA 2007	10	0.21	1.57	0.10	0.00
GUA 2017	16	0.13	1.73	0.09	0.01
FUN 2017	16	0.13	1.33	0.03	0.00
**Mean**	**14**	**0.15**	**1.54**	**0.07**	**0.003**

References

1. Herten, K.; Hestand, M.S.; Vermeesch, J.R.; Van Houdt, J.K. GBSX: a toolkit for experimental design and demultiplexing genotyping by sequencing experiments. *BMC Bioinformatics* **2015**, *16*, 73, doi:10.1186/s12859-015-0514-3.
2. Bushnell, B. *BBMap: a fast, accurate, splice-aware aligner*; Lawrence Berkeley National Lab.(LBNL), Berkeley, CA (United States): 2014.
3. Langmead, B.; Salzberg, S.L. Fast gapped-read alignment with Bowtie 2. *Nat. Meth.* **2012**, *9*, 357.
4. Li, H.; Handsaker, B.; Wysoker, A.; Fennell, T.; Ruan, J.; Homer, N.; Marth, G.; Abecasis, G.; Durbin, R. The sequence alignment/map format and SAMtools. *Bioinformatics* **2009**, *25*, 2078-2079.
5. Korneliussen, T.S.; Albrechtsen, A.; Nielsen, R. ANGSD: analysis of next generation sequencing data. *BMC Bioinformatics* **2014**, *15*, 356, doi:10.1186/s12859-014-0356-4.
